# Ghost cell odontogenic carcinoma: A rare case report and review of literature

**DOI:** 10.1097/MD.0000000000035225

**Published:** 2023-09-22

**Authors:** Yong Xia, Zongchang Song, Xinlei Zhang, Xinhong Guan, Guifang Tan, Yi Le, Shuang Liu, Hui Xue, Jing Li, Yajun Zhang, Jing Chen, Huajuan Jiang, Xia Jiang, Yanxia Cheng, Chuchu Zhou, Xu Sha, Jin-Xin Lou

**Affiliations:** a Department of Oncology, Shanghai University Affiliated Mengchao Cancer Hospital, Shanghai, China; b Department of Radiotherapy, Tongren Hospital Affiliated Shanghai Jiao Tong University School of Medicine, Shanghai, China; c Nuclear Radiation Injury Protection and Treatment Department, Navy Medical Center of PLA, Shanghai, China.

**Keywords:** chemotherapy, ghost cell odontogenic carcinoma, immunotherapy, PD-1, toripalimab

## Abstract

**Rationale:**

Ghost cell odontogenic carcinoma is a rare malignant odontogenic carcinoma characterized by the presence of ghost cells. It has a nonspecific clinical and radiographic presentation and can be locally destructive and invasive, sometimes with distant metastases. However, no effective systemic therapy is currently recommended for such patients.

**Patient concerns:**

The patient has been unable to undergo surgery or radiotherapy again. Therefore, he was referred to our department for a more aggressive, multimodal systematic treatment program.

**Diagnoses:**

The histopathological examination was morphologically suggestive of ghost cell odontogenic carcinomas.

**Interventions:**

We report a case of locally invasive primary inoperable odontogenic shadow cell carcinoma in a 31-year-old Chinese man who achieved treatment with Toripalimab and chemotherapy, followed by Toripalimab maintenance therapy after 6 cycles.

**Outcomes:**

He achieved partial remission after treatment. The quality of life significantly improved after treatment. There were no grade 3/4 treatment-related adverse events during treatment.

**Lessons:**

This case presented that Toripalimab and chemotherapy may be a safe and effective systemic therapy for ghost cell odontogenic carcinoma.

## 1. Introduction

Odontogenic tumors are rare neoplasms that constitute < 1% of the oral tumors.^[1]^ Odontogenic ghost cell lesions comprise calcifying odontogenic cysts, dentinogenic ghost cell tumors and ghost cell odontogenic carcinomas (GCOCs).^[2]^ GCOC is a relatively rare distinct odontogenic neoplasm accounting for 0.23% of all odontogenic tumors.^[3,4]^ with only about 50 cases reported in the English literature so far. The 5-year survival rate for GCOC is about 73% and distant metastases are rare.^[5]^

The recommended treatment for GCOC is extensive surgical excision with clean margins.^[6,7]^ To date, no research has been able to determine whether immunotherapy and chemotherapy are effective in treating inoperable ghost cell odontogenic carcinoma.^[6,8]^ This report presents a rare case of locally invasive primary inoperable GCOC of a 31-year-old Chinese male patient and describes its clinicopathological features, radiological findings, and the treatment along with detailed review of literature.

## 2. Case report

In August 2022, a 31-year-old male patient with locally invasive primary inoperable odontogenic shadow cell carcinoma with pain in the right mandibular region and right orbital region was admitted to the Department of Oncology of our hospital (Shanghai University Affiliated Mengchao Cancer Hospital). He had a history of grade 2 hypertension, and he had no history of long-term drinking, diabetes mellitus, autoimmune diseases, or any other underlying disease.

In June 2021, the patient went to a hospital in Xinjiang for help due to right cheek traumatic injury. The brain magnetic resonance imaging (MRI) examination suggested a cystic lesion in the right maxilla, and the patient underwent a right maxillectomy with clear margins in August 2021. Adjuvant radiotherapy treatment was performed 1 month after surgery. The first histopathological examination was morphologically suggestive of ameloblastic carcinoma.

**Figure 1.**
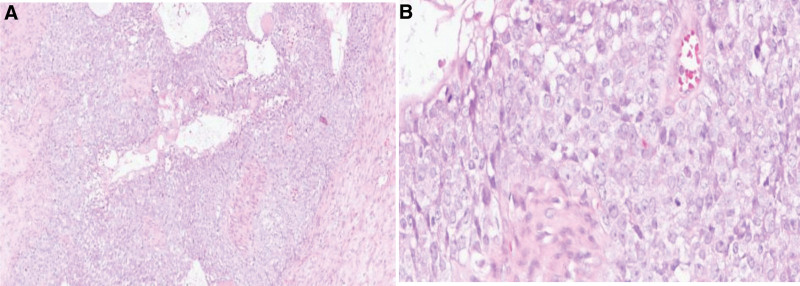
Hematoxylin and eosin (H&E) stained section showing a small number of ghost cells and dysplastic dentin on the surface (A) H&E stain, ×10, (B) H&E stain, ×40.

In March 2022, the patient developed massive nosebleed, and MRI suggested a new tumor lesion in the nasal cavity. In May 2022, the patient underwent endoscopic resection of nasal tumors and local lesions under general anesthesia in a local hospital in Shanghai. The histopathological examination was morphologically suggestive of GCOC (Fig. 1). Under the microscope, a small number of shadow cells and hypoplastic dentin with necrosis, tumor thrombus in the blood vessels, hyperplasia of spindle cells in the stroma with atypia, and nuclear division were observed. In August 2022, sinus MRI showed extensive soft tissue lesion in bilateral nasal cavity and paranasal sinuses, nasopharynx, middle skull base, and bilateral cavernous sinus, and tumor recurrence was considered. In August 15, 2022, palliative resection of paranasal sinus lesion was performed again in Shanghai Hospital, and postoperative pathology again suggested GCOC.

**Figure 2.**
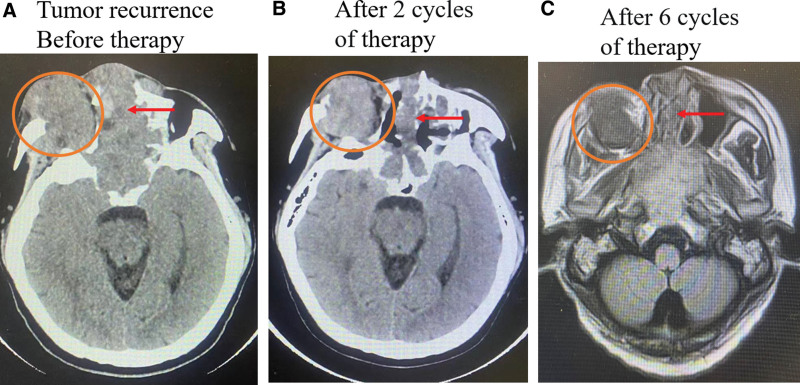
Representative imaging assessments during each period. Head computed tomography (CT) assessment before therapy (A) and after 2 cycles of therapy (B). Head magnetic resonance (T2-FLATR) assessment after 6 cycles of therapy (C). Red arrows indicate the nasal cavity tumor. The orange circles indicate the right orbital tumor.

**Figure 3.**
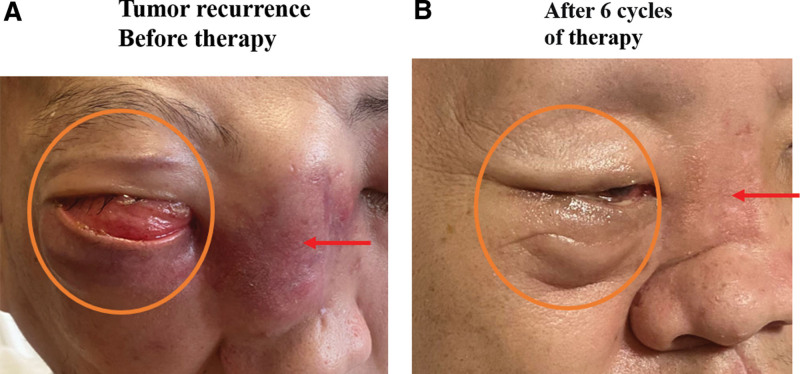
Facial view before therapy (A) and after 6 cycles of therapy (B). Red arrows indicate the nasal bridge tumor. The orange circles indicate the right orbital tumor.

In August 25, 2020, the patient was admitted to the department of oncology of our hospital (Shanghai University Affiliated Mengchao Cancer Hospital) due to inoperable local multiple metastases, severe pain in the right maxilla and visual loss. Beginning in September 1, 2022, He received 240mg of Toripalimab in combination with chemotherapy (albumin-paclitaxel 200 mg/m^2^ and cisplatin 75 mg/m^2^) every 3 weeks for 6 cycles. There was no fever, vomiting, pneumonia, allergy, or any other serious side effects during treatment. After 2 cycles of Toripalimab in combination with chemotherapy therapy, the lesion of nasal cavity significantly shrank, accompanied by a significant improvement in the patient’s pain and nasal congestion. After 3 cycles, the lesion of right orbital significantly shrank, accompanied by a slight recovery of the patient’s light perception (Fig. 2A and B). After 6 cycles, the diameter of right orbital nodule reduced from 53.3 mm to 34.8 mm (reduction of 34.7%) (Fig. 2A and C). Partial response was achieved according to Response Evaluation Criteria in Solid Tumors V.1.1. The facial view also showed significant reduction of the tumor in the bridge of the nose and the right orbit (Fig. 3). The patient then received maintenance treatment with 240 mg of Toripalimab every 3 weeks starting February 7, 2023. We will continue to focus on the patient’s follow-up.

We first reported Toripalimab with chemotherapy (albumin-paclitaxel and cisplatin) that was well effective and low toxic in the treatment of locally invasive primary inoperable odontogenic shadow cell carcinoma.

## 3. Discussion

The GCOC has reported about 50 cases worldwide, and the data show that the average age of patients is about 40 (13–86 years), and most of them are male (male-to-female ratio: 4:1), especially in Asia.^[2,9]^ GCOC are more common in maxilla. The Clinical features of this case are consistent with the previous reports. Table 1 summarizes the previously reported clinical features of GCOC.^[2,10]^

**Table 1:** Clinical features of reported cases of odontogenic ghost cell carcinoma.

Variable	GCOC (only about 50 case)
Average age	40 (13–86 years)
Male-to female ratio	3.4:1
Race/ethnicity	Asian (37.5%) Black (8.3%) White (6.3%) N/A (47.9%)
Main anatomical location affected	Maxilla (especially anterior) (62.5%) Mandible (37.5%)
Main signs and symptoms	Swelling/enlargement (79.2%) Pain (29.2%) Ulceration of the underlying mucosa and/or adjacent skin (18.8%) Bleeding/epistaxis (12.5%)
Main treatment	Radical surgery (45.8%) Radical surgery + neck dissection (8.3%) Radical surgery + radiotherapy (18.8%) Radical surgery + radiotherapy + chemotherapy (4.2%) Conservative surgery (2.1%) Conservative surgery + radiotherapy (2.1%)
Recurrence/metastases	No recurrence (43.8%) High recurrence rate (39.6%) Distant metastasis (14.6%) to: lungs (10.4%), brain/cranial (4.2%).

GCOC = odontogenic ghost cell carcinoma, N/A = not available.

At present, extensive surgical resection is still the main treatment method, and the efficacy of radiotherapy, chemotherapy, immunotherapy and targeted therapy is still uncertain.^[8]^ Besides, immunotherapy has not been reported in locally invasive primary inoperable GCOC. Our case shares clinical similarities with other reports, including a male partiality and maxillary predominance and ethnicity predominance.^[7,11]^ Rapid progression and local recurrence are commonly reported in GCOC. In our case, multiple local metastases occurred in the nasal cavity, right orbit and parotid gland only half a year after surgical resection combined with adjuvant radiotherapy. The rapid recurrence and multiple metastases of GCOC made the patient lose the opportunity of radical resection and radiotherapy from August 2022. We need to find other treatment options to control the progression of GCOC.

Combined immune checkpoint inhibitor (especially Pembrolizumab and Toripalimab) with platinum-based doublet chemotherapy brought about significant improvement in overall survival as the first line treatment in metastatic and recurrent head and neck cancer patients.^[12,13]^ The success of immunotherapy in head and neck cancer gives us hope for effective control of GCOC progression. Pembrolizumab was economically unavailable for this patient, so we recommended Toripalimab to him, a recombinant, humanized programmed death receptor-1 monoclonal antibody that had been approved in recurrent/metastatic nasopharyngeal carcinoma in 2021 in China.^[13,14]^ In this case, according to the chemotherapy regimen of head and neck cancer, we selected the less toxic agents (albumin-paclitaxel that did not require corticosteroid pretreatment combined with cisplatin).^[15]^ After treatment, the patient achieved a clinical partial response, and the quality of life significantly improved. There were no grade 3/4 treatment-related adverse events during treatment.

There are still some regrets in this case, the first one is that the patient failed to achieve clinical complete remission and restore the patient’s vision, so the treatment of locally invasive primary inoperable GCOC patients still has greater challenges. Secondary is the failure to observe progression-free survival, which requires further follow-up. Finally, due to the small number of GCOC cases and the lack of relevant pathological research basis, the relationship between ameloblastic carcinoma and GCOC was not discussed in the cases.

## 4. Conclusion

We suggested that Toripalimab in combination with chemotherapy may be an effective and tolerable approach for patients with locally invasive primary inoperable GCOC.

## Acknowledgments

Thank all the staff authors for their contributions in this study.

## Author contributions

**Conceptualization:** Yong Xia, Zongchang Song, Xu Sha, Jin-Xing Lou.

**Data curation:** Zongchang Song, Xinlei Zhang, Xinhong Guan, Yi Le, Xu Sha.

**Formal analysis:** Guifang Tang, Yi Le, Shuang Liu, Hui Xue, Yajun Zhang.

**Investigation:** Guifang Tang, Yi Le, Shuang Liu, Hui Xue, Jing Li, Yajun Zhang, Jing Chen, Huajuan Jiang, Xia Jiang, Yanxia Cheng, Chuchu Zhou.

**Supervision:** Shuang Liu, Huajuan Jiang, Yanxia Cheng, Chuchu Zhou.

**Writing – original draft:** Yong Xia, Zongchang Song, Xinlei Zhang, Xinhong Guan, Xu Sha.

**Writing – review & editing:** Yong Xia, Jin-Xing Lou.
